# Growth stimulation of *Bifidobacterium* from human colon using daikenchuto in an in vitro model of human intestinal microbiota

**DOI:** 10.1038/s41598-021-84167-z

**Published:** 2021-02-25

**Authors:** Kengo Sasaki, Daisuke Sasaki, Katsunori Sasaki, Yuto Nishidono, Akihiro Yamamori, Ken Tanaka, Akihiko Kondo

**Affiliations:** 1 Graduate School of Science, Technology and Innovation, Kobe University, 1-1 Rokkodai-cho, Nada-ku, Kobe, Hyogo 657-8501 Japan; 2 Sumitomo Chemical, Co., Ltd., 27-1 Shinkawa 2-chome, Chuo-ku, Tokyo, 104-8260 Japan; 3 College of Pharmaceutical Sciences, Ritsumeikan University, 1-1-1 Noji-Higashi, Kusatsu, Shiga 525-8577 Japan; 4 RIKEN Center for Sustainable Resource Science, 1-7-22 Suehiro-cho, Tsurumi-ku, Yokohama, Kanagawa 230-0045 Japan

**Keywords:** Biotechnology, Health care

## Abstract

Daikenchuto (DKT) is a Japanese traditional herbal (Kampo) medicine containing ginseng, processed ginger, and Japanese or Chinese pepper. We aimed to determine how DKT affects human colonic microbiota. An in vitro microbiota model was established using fecal inocula collected from nine healthy volunteers, and each model was found to retain operational taxonomic units similar to the ones in the original human fecal samples. DKT was added to the in vitro microbiota model culture at a concentration of 0.5% by weight. Next-generation sequencing of bacterial 16S rRNA gene revealed a significant increase in the relative abundance of bacteria related to the *Bifidobacterium* genus in the model after incubation with DKT. In pure cultures, DKT significantly promoted the growth of *Bifidobacterium adolescentis*, but not that of *Fusobacterium nucleatum* or *Escherichia coli*. Additionally, in pure cultures, *B. adolescentis* transformed ginsenoside Rc to Rd, which was then probably utilized for its growth. Our study reveals the in vitro bifidogenic effect of DKT that likely contributes to its beneficial effects on the human colon.

## Introduction

Daikenchuto (DKT) is a traditional herbal (Kampo) medicine in Japan that comprises three medicinal herbs: ginseng (*Panax ginseng*), Japanese pepper (*Zanthoxylum piperitum*) or Chinese pepper (*Zanthoxylum bungeanum*), and processed ginger (*Zingiber officinale*)^1^. DKT is prepared by mixing the herbs, followed by extraction using hot water and finally converting the extract into a powder or soft form^1^. Treatment with DKT alleviated gastrointestinal dysmotility by increasing defecation frequency and decreasing bowel gas in patients after a total gastrectomy^2,3^. DKT exerted an anti-inflammatory effect in a mouse post-operative ileus model^4^. Inoue et al.^5^ reported that DKT reduced intestinal fibrosis in a rat model of Crohn’s disease. The constituents of DKT are absorbed and/or metabolized at different rates^1^. The main active constituent of Japanese pepper, hydroxysanshool, is rapidly absorbed before reaching the colon. The main active compounds of processed ginger, [6]-shogaol and [6]-gingerol (6G), are metabolized in the upper small intestine and liver. In contrast, most of the ginseng compounds reach the colon intact, and their hydrophilic constituents, such as ginsenosides, are absorbed only after they are metabolized to hydrophobic metabolites by the gut microbiota^6^. A previous study examined the effect of DKT on the gut microbiota in mice and reported an increase in the abundance of the probiotic strain, *Lactococcus lactis*^7^*.* However, it is unclear how DKT affects human colonic microbiota.

The gastrointestinal tract microbiota, a multispecies microbial community, has been shown to interact with the host mutually and essential for maintaining host health^8^. The microbial cell density increases steadily along the gastrointestinal tract, with the levels being low in the stomach and very high in the colon^9^. An in vitro model of the human colonic microbiota was used to test microbial responses to various agents and stimuli, and its major advantages were reproducibility, limited ethical restraints, and shorter duration of study compared to human clinical trials or animal trials^10^. Previously, we reported an in vitro human colonic microbiota model that could retain bacterial species diversity of fecal inoculum (hereafter referred to as Kobe University Human Intestinal Microbiota Model, KUHIMM)^11^. We simulated the response of human colonic microbiota to dietary prebiotics using this model in combination with next-generation sequencing for 16S rRNA gene amplicon analysis^12^.

In this study, we assessed the effect of 0.5% wt. DKT on human colonic microbiota in our in vitro model, KUHIMM. The bacterial composition in KUHIMM was determined following DKT administration and compared to that in the absence of DKT. In addition, we examined the effects of DKT administration on each representative type of colonic bacteria.

## Results

### Increase in the abundance of *Bifidobacterium* following administration of DKT to KUHIMM

An in vitro human colonic microbiota model, KUHIMM, was established using each of the nine human fecal samples as inoculum. We added 0.5% wt. DKT to KUHIMM to examine its effects on the microbiota. In all KUHIMM samples, eubacterial copy numbers were initially 2.41 ± 3.76 × 10^8^ copies/mL and reached 3.45 ± 2.42 × 10^11^ copies/mL at 48 h of cultivation.

Next-generation sequencing was used for bacterial 16S rRNA gene sequence analysis and yielded 4,776,828 quality reads for bacteria from the original fecal samples and KUHIMM cultures with and without DKT (Table 1 and Supplementary Figure S1). The corresponding KUHIMMs, with and without DKT, harbored similar numbers of operational taxonomic units (OTUs) as those in fecal inoculums (Mann–Whitney *U*-test, *P* = 0.19 and 0.93, respectively). Moreover, the Chao1 richness estimator indicated no significant differences between fecal inoculums and corresponding KUHIMMs with and without DKT (Mann–Whitney *U*-test, *P* = 0.29 and 0.72, respectively). Bacterial community diversity indices (Shannon and Inverse Simpson) were lower in the corresponding KUHIMMs without DKT than in fecal inoculums (Mann–Whitney *U*-test, *P* = 0.027 and 0.042, respectively). However, no significant differences were observed in the Shannon and Inverse Simpson indices of KUHIMMs with DKT and without DKT (Mann–Whitney *U*-test, *P* = 0.29 and 0.38, respectively).

**Table 1:** Summary of bacterial 16S rRNA gene sequencing data and alpha diversity measures.

	Feces (n = 9)	KUHIMM	
		Culture (n = 9)	Culture + DKT (n = 9)
Read counts	171,268 ± 37,246	185,402 ± 25,869	174,089 ± 48,768
Observed OTUs	1429 ± 382	1388 ± 252	1205 ± 336
Chao 1	3485 ± 560	3493 ± 783	3283 ± 964
Shannon	6.070 ± 0.436	5.435 ± 0.586*	5.236 ± 0.562*
Inverse Simpson	1.044 ± 0.013	1.077 ± 0.035*	1.092 ± 0.038*

The values show the mean ± standard deviation. The asterisks (*) indicate a significant difference (*P < 0.05 by Mann–Whitney’s *U* test) between the microbiota in original feces and corresponding KUHIMMs with and without daikenchuto (DKT).

*OTUs* operational taxonomic units.

**Figure 1:**
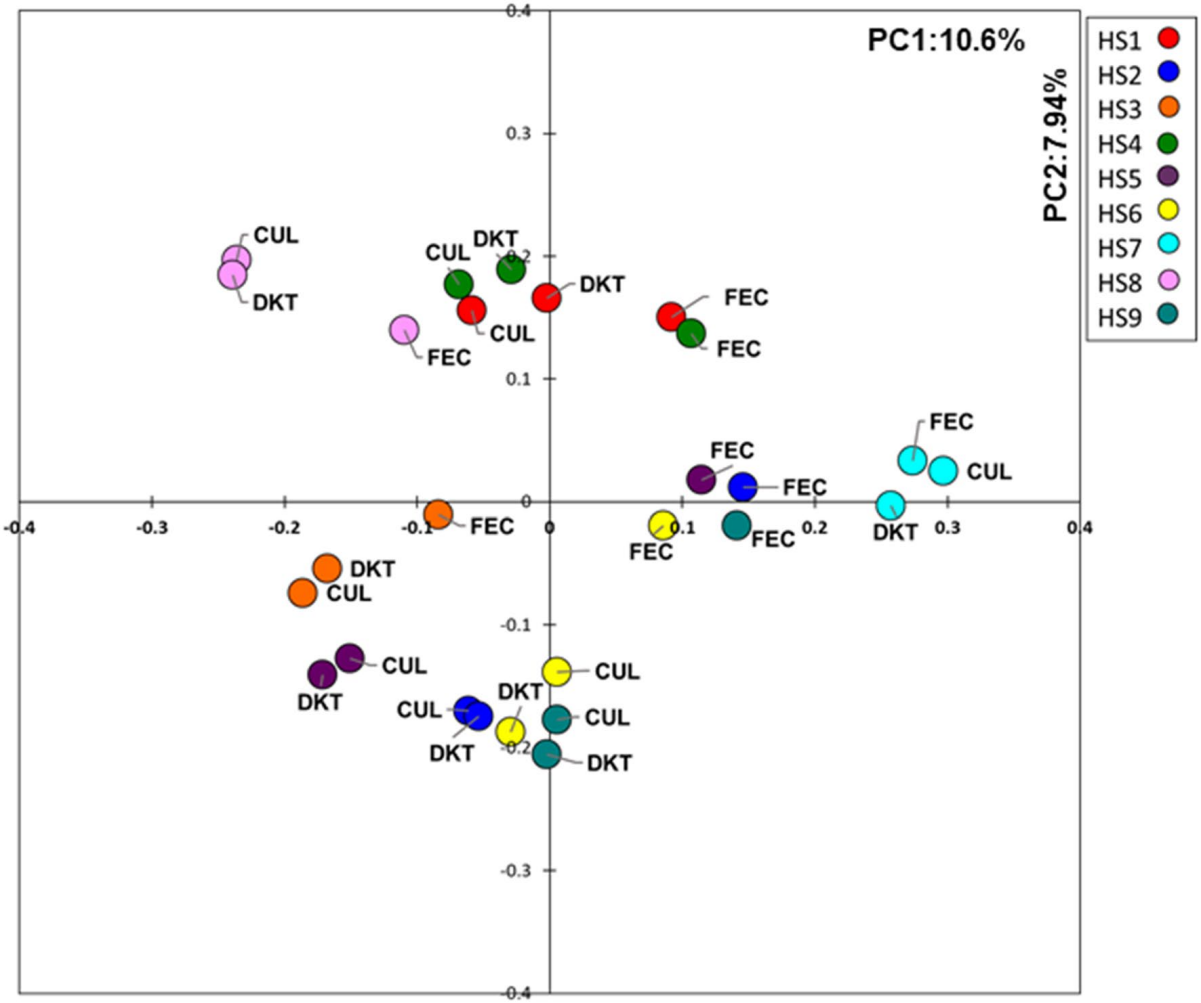
Clustering of microbiota with principal coordinate analysis using UniFrac. *FEC* fecal inoculums; *CUL* in vitro human colonic microbiota model (KUHIMM) culture without daikenchuto (DKT) after 48 h of fermentation; *DKT* KUHIMM culture with DKT.

**Figure 2:**
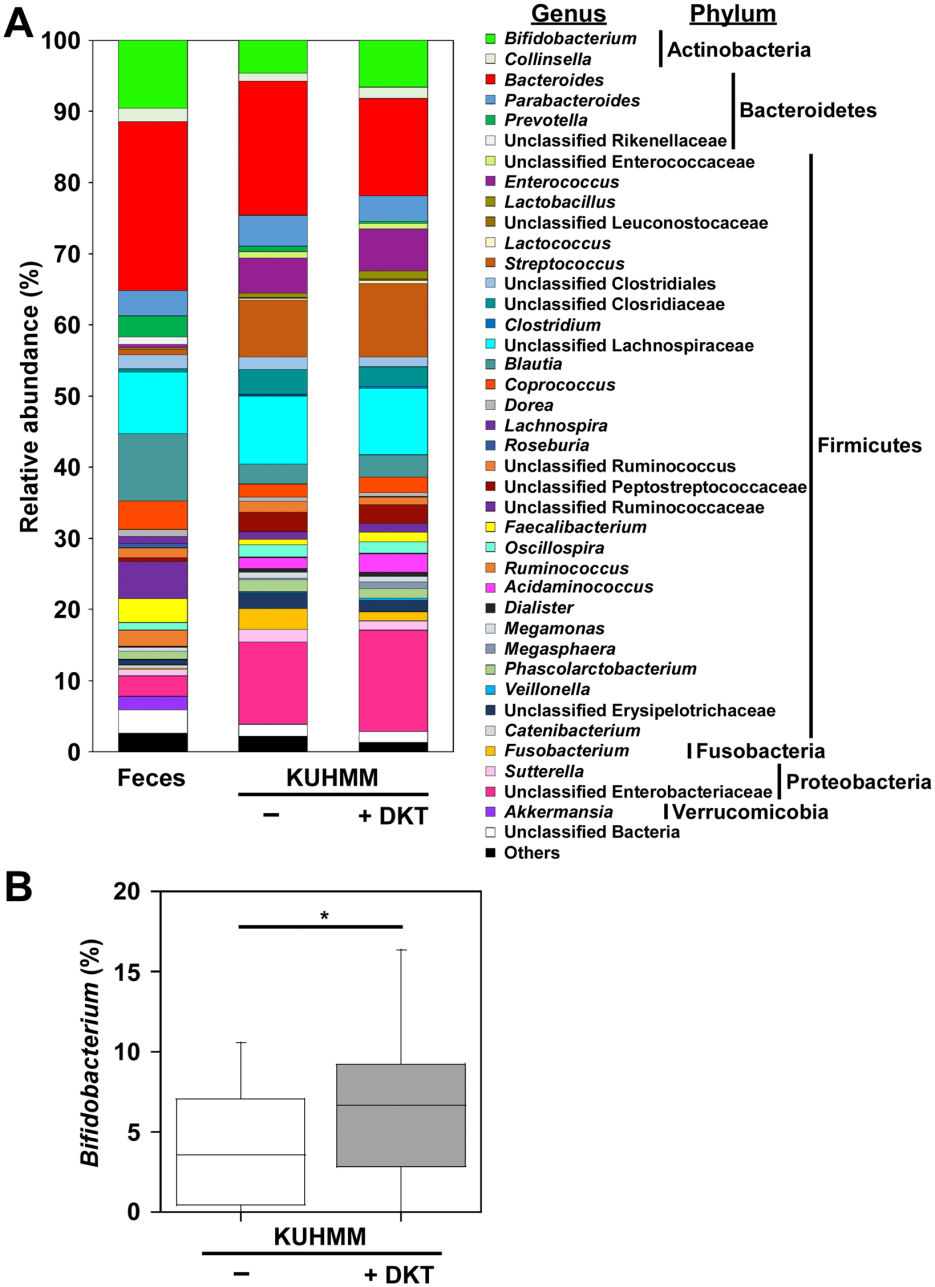
Effect of the daikenchuto (DKT) on bacterial taxonomic composition. (**A**) Genus-level classification of bacteria in original fecal inoculums (Feces) and in vitro human colonic microbiota model (KUHIMM) without (−) and with DKT (+ DKT) after 48 h of fermentation. Genera of lower abundance (< 1.0%) and lower similarity (< 97%) were included as Others and Unclassified Bacteria, respectively. Means are shown for nine healthy human subjects. (**B**) Relative abundances of bacteria related to genus *Bifidobacterium*. *P < 0.05.

Principal coordinate analysis revealed that DKT administration did not have such impact on total microbiota structure in the KUHIMM culture (Fig. 1). Figure 2 shows the mean bacterial genus-level distribution in the original fecal inocula and the corresponding KUHIMMs, with and without DKT, for the nine healthy human subjects. Most of the bacterial genera found in the human fecal samples belonged to the phyla *Actinobacteria*, *Bacteroidetes*, *Firmicutes*, *Fusobacteria*, *Proteobacteria*, and *Verrucomicrobia*; these phyla were also found in the KUHIMMs (Fig. 2A). Interestingly, the relative abundance of bacteria related to the *Bifidobacterium* genus was significantly higher in the KUHIMMs with DKT than in the KUHIMMs without DKT (Wilcoxon signed-rank test, *P* = 0.020, Fig. 2B).

### Direct effects of DKT on gut microbiota

**Figure 3:**
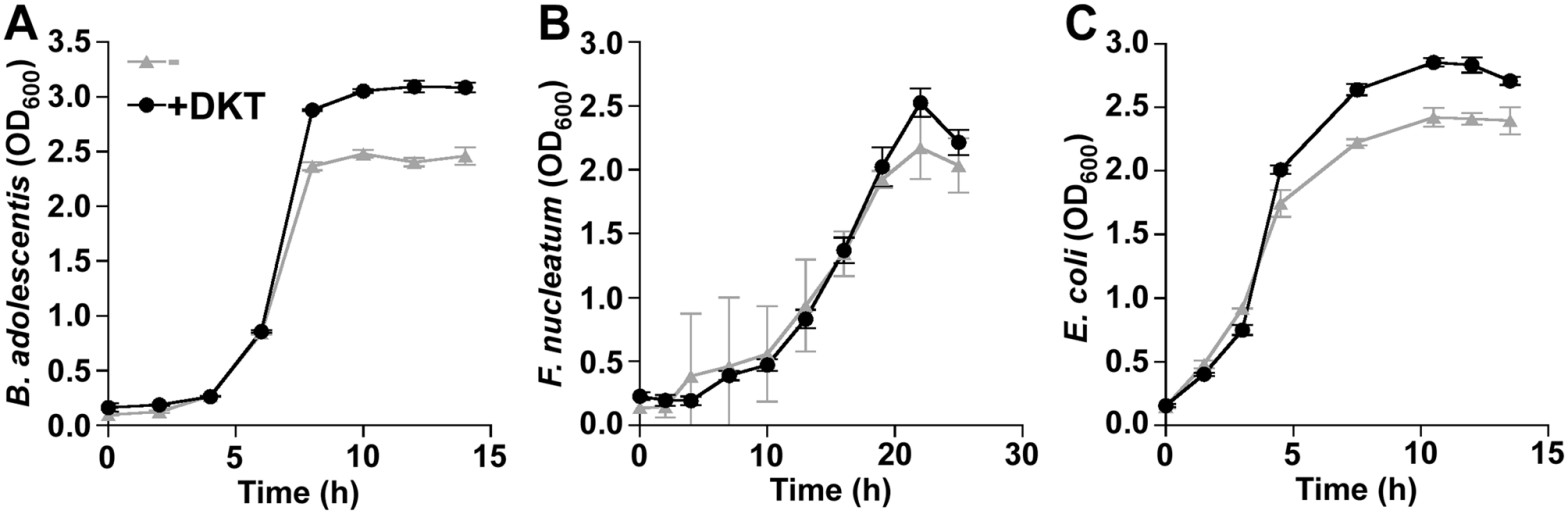
Growth of each studied bacterial species in the presence ( +) and absence (−) of daikenchuto (DKT). **(A)** *Bifidobacterium adolescentis*, **(B)** *Fusobacterium nucleatum*, and **(C)** *Escherichia coli*.

To directly investigate how 0.5% wt. DKT affects microbial growth, we cultured each bacterial species, *Bifidobacterium adolescentis*, *Fusobacterium nucleatum*, and *Escherichia coli*, with and without DKT. DKT significantly promoted the growth of *B. adolescentis* in pure cultures (Fig. 3A, P = 0.024, two-way analysis of variance (ANOVA) with repeated measurements). However, DKT did not significantly promote the growth of *F. nucleatum* or *E. coli* (Fig. 3B,C, P = 0.62 and 0.052, respectively, two-way ANOVA with repeated measurements).

Next, we measured the concentrations of DKT constituents before and after cultivation with each bacterial species (Table 2). First, we measured the concentrations of 0.5% wt. DKT before cultivation and compared these concentrations to those after cultivation with *B. adolescentis*, *F. nucleatum*, or *E. coli*. Interestingly, although ginsenoside Rc concentration was lower after cultivation with *B. adolescentis* (*P* = 0.0005, Student’s *t*-test), it did not change after cultivation with *F. nucleatum* or *E. coli* (*P* = 0.083 and 0.10, respectively, Student’s *t*-test). Thus, *B. adolescentis* metabolized Rc to a greater extent than did *F. nucleatum* and *E. coli*. After cultivation with *B. adolescentis*, ginsenoside Rd concentration was higher than in the original DKT (*P* = 0.0002, Student’s *t*-test), but lower after cultivation with *F. nucleatum* or *E. coli* (*P* = 0.0061 and 0.0026, respectively, Student’s *t*-test). Thus, *B. adolescentis* produced more ginsenoside Rd than did *F. nucleatum* and *E. coli*.

**Table 2:** Concentrations (μg/mL) of constituents of daikenchuto (DKT).

Constituent	0.5% wt. DKT	+ *B. adolescentis*	+ *F. nucleatum*	+ *E. coli*
**Ginsenoside**				
Rb1	9.31 ± 0.78	8.53 ± 0.01	8.45 ± 0.31	8.78 ± 0.10
Rb2	5.77 ± 0.44	4.55 ± 0.11	4.65 ± 0.18	4.95 ± 0.44
Rc	3.84 ± 0.23	1.07 ± 0.15	3.29 ± 0.07	3.26 ± 0.16
Rd	3.22 ± 0.11	5.08 ± 0.09	2.30 ± 0.13	2.42 ± 0.04
Re	4.23 ± 0.07	4.01 ± 0.06	3.75 ± 0.07	3.85 ± 0.08
Rg1	11.03 ± 0.38	9.54 ± 0.12	9.42 ± 0.11	10.07 ± 0.40
Hydroxysanshool	6.84 ± 0.44	3.77 ± 0.08	1.47 ± 0.19	1.34 ± 0.28
**Gingerol**				
6G	2.63 ± 0.30	3.26 ± 0.15	2.13 ± 0.32	3.08 ± 0.03

Data are presented as mean ± SD. Concentrations are shown for 0.5% wt. DKT before cultivation and after cultivation with *Bifidobacterium adolescentis*, *Fusobacterium nucleatum*, or *Escherichia coli.*

## Discussion

In this study, we incubated fecal samples with 0.5% wt. DKT in a model culture system. In this system comprising many human fecal microbiota samples, we observed a significant increase in the relative abundance of the *Bifidobacterium* genus. This observation was supported by our finding that DKT increased the growth of *B. adolescentis *in vitro using pure cultures. *B. adolescentis* strains represent key taxa of an adult-associated bifidobacteria because they appear to specifically colonize the guts of adult individuals^13^. Health-promoting probiotic properties have been associated with some bifidobacterial strains belonging to the species, *B. adolescentis*, *B. animalis*, *B. bifidum*, *B. breve*, and *B. longum*^14^. Thus, DKT can be considered a prebiotic that stimulates the growth of beneficial bacteria such as *B. adolescentis*. Oral supplementation with *B. adolescentis* protected against the development of nonalcoholic steatohepatitis in a mouse model^15^. Furthermore, anti-hepatitis B viral activity has been reported for *B. adolescentis*^16^.

We found that *B. adolescentis* converted ginsenoside Rc into Rd. Biotransformation of ginsenoside Rc into Rd by α-_L_-arabinofuranosidase has been reported in *Bifidobacterium longum* H-1^17^ and *Leuconostoc* sp. 22–3^18^. Further, Suzuki et al.^19^ identified α-_L_-arabinofuranosidase enzyme activity in *B. adolescentis*. Substrates of the α-_L_-arabinofuranosidase enzyme include the terminal residues of 1,5-α-_L_-arabinan and 1,5-α-_L_-arabino-oligosaccharide and the branched arabinofuranoside residues of arabinoxylan and arabinan^19^. Thus, α-_L_-arabinofuranosidase in *B. adolescentis* in this study and the gut could catalyze the hydrolysis of the arabinofuranoside moiety attached to ginsenoside Rc^18^. Arabinofuranose is expected to be transported into the cell via transporters and utilized via arabinose utilization pathways^20^. *B. longum* subsp. *longum* utilizes arabinose as a carbon source^21^. Compound K, which is efficiently produced from ginsenoside Rd to a greater extent than from Rc, Rb1, and Rb2 and is absorbed by various target cells, has recently attracted a great deal of attention because of its anti-tumor, anti-inflammatory, anti-allergic, and hepatoprotective effects^1,22^. The above effects of compound K could be attributed to the stimulation of the growth of bifidobacteria.

DKT was added at a concentration of 0.5% wt. to the in vitro human colonic microbiota model, KUHIMM, which has a working volume of 100 mL. Colon content has been estimated to be approximately 400 mL^23^. Thus, we concluded that 2 g (= 0.5 × 4) of DKT is required to exert its bifidogenic effect in the colon. This amount of DKT is comparable with the amount previously used in human clinical studies (2.5–7.5 g-DKT/day)^24^. A limitation of this study may be the relatively small sample size of human subjects. In addition, the in vitro human colonic microbiota model, KUHIMM, lacks host factors, and improvement should be considered in future experiments.

## Methods

### Fecal specimen collection

Fecal samples were obtained from nine healthy Japanese volunteers, who had not been treated with antibiotics for more than 6 months prior to the experiment. All participants were recruited according to the inclusion criteria: age of 30 to 60 years, being Japanese, non-smoking status, and good health and physical condition (Supplementary Table S1). All subjects provided written informed consent prior to specimen collection. Immediately following collection, each fecal sample was stored in an anaerobic culture swab (212550 BD BBL Culture Swab; Becton, Dickinson and Company, New Jersey, USA) and used within 24 h. The study was performed in accordance with the guidelines of Kobe University Hospital, and approved by the institutional ethics review board of Kobe University. All methods in this study were in accordance with the principles of the Declaration of Helsinki.

### Operation of the model culture system with and without DKT

We used a small-scale multi-channel fermentor (Bio Jr.8, ABLE, Tokyo, Japan) composed of eight parallel and independent anaerobic culturing vessels, as described by Sasaki et al.^12^. Each vessel contained autoclaved (100 mL, 115 °C for 15 min) Gifu anaerobic medium [GAM (Code 05422); Nissui Pharmaceutical Co, Tokyo, Japan], with the initial pH adjusted to 6.5. Anaerobic conditions in the vessel were achieved by purging with a mixture of N_2_ and CO_2_ (80:20, 15 mL/min) that was filter-sterilized through a 0.2-μm Polytetrafluoroethylene Membrane Filter (Pall Corporation, Port Washington, NY, USA) at 37 °C for 1 h prior to cultivation. To prepare the inoculum, the fecal sample in the swab was suspended in phosphate buffer (0.1 M, 2.0 mL, pH 6.5, consisting of 61.65:28.35 mixture of NaH_2_PO_4_ and Na_2_HPO_4_) supplemented with L-ascorbic acid (1.0% w/v; Wako Pure Chemical Industries, Osaka, Japan).

Cultivations were initiated by inoculating one fecal suspension (100 μL) into each vessel to construct the in vitro human colonic microbiota model, KUHIMM. During fermentation at 37 °C, the culture broth was stirred at 300 rpm with a magnetic stirrer and continuously purged with a filter-sterilized gas mixture. Aliquots of culture broth were collected from the vessel at 48 h after the initiation of cultivation. Feces and culture broth samples were stored at – 20 °C until use.

DKT used in this study was supplied by Matsuura Yakugyo Co., Ltd. (Aichi, Japan). It was a brown, soft extract, 1.25 g of which was produced from 1 g of Zanthoxylum peel, 2 g of processed ginger, and 1 g of ginseng. Unlike the Kampo drug generally known as Daikenchuto, this DKT extract did not include starch syrup, which mainly consists of maltose and other sugars, to avoid the starch syrup effect on the growth of gut microbiota. To determine the effect of DKT, it was added into one of the vessels at a final concentration of 5.0 g/L (0.5% wt. per 100-mL vessel) prior to cultivation. Additionally, a control vessel was prepared without DKT.

### 16S rRNA bacterial profiling

Microbial genomic DNA was extracted from suspended feces and culture broth obtained from KUHIMM. Purified DNA was eluted into TE buffer (10 mM Tris-HCl, 1.0 mM EDTA) and stored at – 20 °C until use. Bacterial 16S rRNA genes (V3‒V4 region) were amplified using genomic DNA as the template and primers S-D-Bact-0341-b-S-17 (5′-CCTACGGGNGGCWGCAG-3′) and S-D-Bact-0785-a-A-21 (5′-GACTACHVGGGTATCTAATCC-3′)^25^, as previously described^12^. The libraries were generated using a Nextera kit (Nextera XT Index Kit; Illumina Inc., San Diego, CA, USA). Paired-end sequencing reactions were performed on a MiSeq platform (Illumina). Paired-end reads with quality scores ≥ 20 were joined using Quantitative Insights Into Microbial Ecology (QIIME) version 1.9.1 software^26^. The UCLUST algorithm was used to cluster filtered sequences into OTUs based on a 97% similarity threshold^27^. Chimeric sequences were identified and excluded from the library using USEARCH^28^. Paired-end reads were taxonomically classified via the Greengenes taxonomic database using the Ribosomal Database Project Classifier^29^.

### Real-time PCR analysis

Real-time PCR was performed using LightCycler 96 (Roche Diagnostics GmBH, Germany). The FastStart SYBR Green master mix kit (Roche) was used for reactions with a primer set targeting all eubacteria^30^. The PCR reaction and amplification were performed as described previously^30^.

### Culture of strains

*Bifidobacterium adolescentis* JCM 1275, *Fusobacterium nucleatum* JCM 8532 and *Escherichia coli* JCM 1649 were obtained from the Japan Collection of Microorganisms (JCM). Each strain was cultured in triplicate in GAM at 37 °C under anaerobic conditions (20% CO_2_ and 80% N_2_). For comparison, each strain was cultured similarly by adding DKT (5.0 g/L, corresponding to 0.5% wt.).

### Analysis of DKT constituent

GAM (1 mL) containing DKT (5.0 g/L) and cultures of the above three strains were lyophilized using an FZ-2.5 Labconco vacuum freeze-drier (Asahi Life Science Co. Ltd., Saitama, Japan) after centrifugation at 6000 rpm for 5 min to remove microorganisms. All standard ginsenosides (except ginsenoside Rc), [6]-gingerol and hydroxysanshool were isolated and identified by comparing their spectral data with previously reported data^31–33^. Lyophilized samples were individually dissolved in methanol (1 mL). Each solution was filtered through a 0.45 μm Millipore filter unit (Advantec, Tokyo, Japan), and the filtrate samples (1 μL) were injected into the LC–MS system for analysis. The LC–MS analyses were performed using a Shimadzu LC-IT-TOF mass spectrometer (Shimadzu, Kyoto, Japan) equipped with an ESI interface. The ESI parameters were as follows: source voltage, + 4.5 kV (positive ion mode) and –3.5 kV (negative ion mode); capillary temperature, 200 °C; nebulizer gas flow rate, 1.5 L/min. The mass spectrometer was operated in the positive and negative ion modes, scanning from m/z 150 to 1500. A Waters Atlantis T3 column (internal diameter 2.1 mm × 150 mm, 5 μm) was used, and the column temperature was maintained at 40 °C. The mobile phase was a binary eluent consisting of (A) 5 mM (NH_4_)OAc solution and (B) CH_3_CN under the following gradient conditions: 0–30 min, linear gradient from 10 to 100% B, 30–40 min, isocratic at 100% B. The flow rate was 0.2 mL/min. To quantitate the compounds, the following ions were monitored in the mass chromatograms: ginsenoside Rb1, 583.31 (M + CH_3_COOH-2H)^2−^; ginsenoside Rb2, 568.30 (M + CH_3_COOH-2H)^2−^; ginsenoside Rc, 568.30 (M + CH_3_COOH-2H)^2−^; ginsenoside Rd, 532.29 (M + 2CH_3_COOH-2H)^2−^; ginsenoside Re, 532.29 (M + 2CH_3_COOH-2H)^2−^; ginsenoside Rg1, 459.26 (M + 2CH_3_COOH-2H)^2−^; ginsenoside Rh1, 697.45 (M + CH_3_COOH-H)^−^; sanshool, 248.20 (M + H)^+^; hydroxysanshool, 264.20 (M + H)^+^; and [6]-gingerol, 293.18 (M − H).

### Bioinformatics and statistical analysis

The Chao 1, Shannon, and Inverse Simpson indices were calculated using the QIIME software package (Caporaso et al. 2010). Data were compared between the groups using the Mann–Whitney *U* test, Wilcoxon signed-rank test, or a two-way ANOVA with repeated measurements in the JMP version 12. *P* < 0.05 was considered statistically significant.

## Supplementary Information

Supplementary Information.

## Data Availability

All sequences from the original fecal samples and corresponding KUHIMMs were deposited in MG-RAST as “Model Culture System of Human Colonic Microbiota_Daikenchuto” under the accession numbers mgm4891148.3–mgm4891174.3.
