# Supplementary material for: Growth stimulation of Bifidobacterium from human colon using daikenchuto in an in vitro model of human intestinal microbiota
Source: Sci Rep. 2021 Feb 25;11:4580. doi: 10.1038/s41598-021-84167-z (PMC7907203; doi:10.1038/s41598-021-84167-z)
Supplement: Supplementary file 1 — Supplementary Information. [file 41598_2021_84167_MOESM1_ESM.docx]

**Supporting Information**

**Title**

Growth stimulation of *Bifidobacterium* from human colon using *daikenchuto* in an *in vitro* model of human intestinal microbiota

**Authors**

Kengo Sasaki^1*^, Daisuke Sasaki^1^, Katsunori Sasaki^2^, Yuto Nishidono^3^, Akihiro Yamamori^2^, Ken Tanaka^3^, Akihiko Kondo^1,4^

^1^Graduate School of Science, Technology and Innovation, Kobe University, 1-1 Rokkodai-cho, Nada-ku, Kobe, Hyogo 657-8501, Japan

^2^Sumitomo Chemical, Co., Ltd., 27-1 Shinkawa 2-chome, Chuo-ku, Tokyo 104-8260, Japan

^3^College of Pharmaceutical Sciences, Ritsumeikan University, 1-1-1 Noji-Higashi, Kusatsu, Shiga 525-8577, Japan

^4^RIKEN Center for Sustainable Resource Science, 1-7-22 Suehiro-cho, Tsurumi-ku, Yokohama, Kanagawa 230-0045, Japan

*Corresponding author

Kengo Sasaki

Graduate School of Science, Technology and Innovation, Kobe University, 1-1 Rokkodai-cho, Nada-ku, Kobe, Hyogo 657-8501, Japan

+81-78-803-6462 (TEL/FAX)

[sikengo@people.kobe-u.ac.jp](mailto:sikengo@people.kobe-u.ac.jp)

Supplementary Table S1. Characteristics of study volunteers

|  | **Healthy subject (n = 9)** |
| --- | --- |
| **Age in years (mean ± standard deviation)** | 41.6 ± 7.4 |
| **Sex (Female/Male)** | 4/5 |
| **Race** |  |
| **Asian** | 100% |


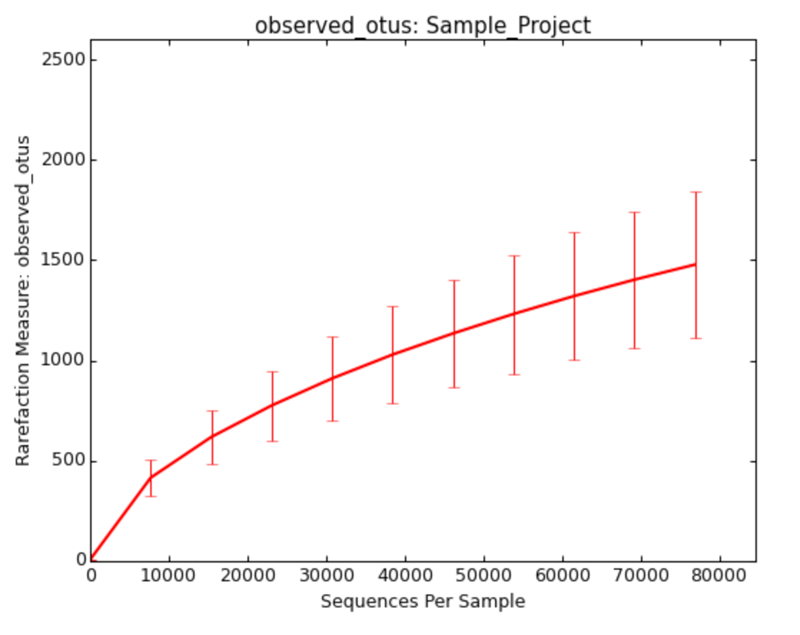


**Supplementary Figure S1. Rarefaction curve of the number of observed OTUs from 16S rRNA sequence data.**
